# Hospital Security Team Involvement in Emergency Mental Health Care of Patients

**DOI:** 10.1001/jamanetworkopen.2025.30439

**Published:** 2025-08-29

**Authors:** Lauren T. Southerland, Adrienne S. Beck, Ellie K. Ganz, Cassandra L. Pasadyn, Steven Wollenberg, Henry W. Young

**Affiliations:** 1 Department of Emergency Medicine, The Ohio State University Wexner Medical Center, Columbus; 2 The Ohio State University College of Medicine, Columbus; 3 Department of Public Safety, The Ohio State University Wexner Medical Center, Columbus

## Abstract

**Importance:**

Hospital-based security teams assist emergency department (ED) staff when there is a risk to staff or patient safety.

**Objective:**

To describe the current practice of hospital security assistance during emergency mental health care.

**Design, Setting, and Participants:**

This qualitative study with mixed methods was a secondary analysis of a retrospective cohort study of patients in the ED for mental health concerns who received involuntary sedation from 2020 to 2021. Any ED note documenting security presence or calls was abstracted as a text snippet. Hospital security data logs were also queried for the same time frame and summarized. Data were analyzed from February to November 2024.

**Main Outcomes and Measures:**

Outcomes included type and quantity of hospital security calls to the ED. A deductive quality analysis was performed with the goal of assessing current clinical documentation practices as an initial step toward improvement. The deductive coding of text snippets began with 10 codes, including verbal threats, physical aggression, and elopement.

**Results:**

Over the 2 years, hospital security had 9288 clinical support encounters in the ED (mean, 12.7 encounters per day). The parent electronic health record abstraction study included 334 patients, of which 244 patients (73.1%) had hospital security involved in their care and so were included in this analysis (146 men [59.8%]; mean [SD] age, 34.5 [13.6] years). Among the study subset, 135 patients (55.3%) were brought to the ED by police. Security was most often called for physical violence (82 patients [33.6%]). De-escalation attempts occurred by ED staff for 29 patients (11.9%) and security personnel for 17 patients (7.0%). Theme 1 was safety: patient-on-patient violence was a new finding: “...this patient stood up out of her chair, ran over to another patient and started punching another patient.” Theme 2 was patients who are unwilling or unable to adhere to the care plan. Disagreement with disposition plans or medical care was a frequent trigger of violence. Theme 3 was that de-escalation attempts were not well documented. “Security called to bedside… [nurse] able to talk patient down at this time.” Theme 4 included legal issues: security assisted with the disposal of illicit substances and use of security body cameras to document patient belongings.

**Conclusions:**

In this study, security teams contributed to the safety of ED staff and patients. This single-center study’s findings suggest the need for future improvements, including improving the documentation of de-escalation techniques used and developing better ways to reduce violence from care plan disagreements.

## Key Points

**Question:**

How are hospital security teams used during emergency mental health care?

**Findings:**

In this qualitative study of hospital security logs and notes from clinical care among 244 patients, hospital security teams contributed to patient and staff safety, elopements or lack of capacity to refuse care, and legal issues, such as illicit substance disposal and care of patient belongings. De-escalation attempts in situations of violence were not well documented in clinical notes.

**Meaning:**

This study found that hospital security teams assisted frequently during emergency mental health care, including in aggressive or violent events and with nonaggression care needs.

## Teaser

This qualitative study explores how hospital security teams are used among patients in the emergency department with mental health concerns.

## Introduction

Emergency departments (EDs) are a medical safety net for all patients, especially those with acute decompensated mental health disorders, substance intoxication, or behavioral concerns.^1,2^ These patients sometimes cannot control their impulses. They may experience paranoia or be easily frightened, leading to aggression and agitation and ultimately harm to the patient or ED staff. Unfortunately, workplace violence in the ED is increasing.^3,4^ Even with violence interventions in place, EDs see rates of 1 to 2 violent patient-on-staff assaults per month (considered type II violence, where the perpetrator is a customer or client).^5^ More than 75% of ED physicians and nurses have faced violence from patients.^6,7^

In response to this growing phenomenon, more EDs are partnering with hospital security or local police forces.^8^ Hospital security guards are trained to de-escalate and safely restrain agitated patients. These personnel can also improve safety by checking patients and visitors for weapons, protecting patient or hospital property, and monitoring public visitation.^9^ However, security teams in hospitals can have downsides, including mistrust among patients, concerns about reducing the psychological safety of patients, and the possibility of excessive restraint use.^10^ One survey found that most ED nurses wanted to reduce the need for security guards, restraints, and involuntary sedation but still found these methods to be necessary for agitated patients.^11^

An Australian hospital reported^12^ 1.8 aggressive patient events requiring security per day, and a qualitative study in India^13^ found that violence more frequently came from patient visitors than patients themselves. However, to our knowledge, there are no previous reports of how hospital security teams contribute to patient care in US hospitals, which may be different. Our goal was to qualitatively evaluate the use of hospital security for patients receiving mental health care in a large US ED. This study reports the assessment of current practice in our hospital and is the start of efforts to identify potential areas for improvement.

## Methods

### Study Description

The setting for this qualitative study was an urban academic health system with a university hospital and a community hospital. The parent study was a retrospective cohort study of patients older than 12 years in the ED for mental health reasons who received involuntary sedation during their ED visit between January 1, 2020, and December 31, 2021. This study was approved by The Ohio State University institutional review board,^14^ which waived the requirement for informed consent due to minimal risk research on data collected from medical records. Study conduct and reporting follow the Consolidated Criteria for Reporting Qualitative Research (COREQ) reporting guideline. This analysis consists of a qualitative evaluation of patients in the parent study who had hospital security teams involved in their care and a parallel quantitative evaluation of hospital security logs. Qualitative and quantitative analyses were done independently on different datasets and then triangulated for final conclusions.

### Data Sources

Data sources included hospital security data logs and hospital electronic health records (EHRs). The hospital security database is an electronic database separate from the hospital EHR, and the data included were from the university ED only. Security data logs were queried for the number and type of encounters during the 2-year study timeline. These categorizations are input by hospital security team members who respond to a call or incident (category definitions are in Table 1). This is a secondary analysis of data abstraction from EHRs, and the development of the dataset was previously reported.^14^ Any ED notes that referred to calling security or the presence of security guards were abstracted as a quantitative (yes or no) variable and an open-text response. For the open-text response, abstractors (L.T.S., A.S.B., E.K.G., and C.L.P.) were instructed to copy any phrases or sentences in notes from the clinical team (eg, nurses, doctors, patient care technicians, or social workers) that described why security was called or referred to the presence of the security team. Abstractors were told that it was a quality check for the quantitative variable and were blinded to the purpose of this secondary analysis. The data they chose to abstract were therefore not biased toward coding specific incidents or items. Race and ethnicity are reported to state the demographic makeup of the population studied. Race and ethnicity were collected from the EHR. Patients in our system are able to self-report, including the categories of American Indian or Alaskan Native, Asian American, Black or African American, White, more than 1 race, and other, which included Middle Eastern, Nepali, Somali, and unknown.

**Table 1.** Categorization of Clinical Encounters in the Emergency Department From Hospital Security Logs

Category code	Encounters, No. (%)	Definition
Standby	5494 (59.2)	Security was requested to stand by in the area due to staff’s concern that a situation may escalate. No physical action was taken.
Nonadherent	2688 (28.9)	The patient was displaying uncooperative behavior, such as not following staff’s directions, or the patient may have just been confused.
Eloping	262 (2.8)	The patient eloped or was in the process of eloping before being stopped.
Verbally aggressive	149 (1.6)	The patient was verbally inappropriate but nothing that was threatening in nature.
Threats made	18 (0.2)	A patient made threats of physical harm to staff, visitors, or patients.
Restraint-medical	107 (1.2)	Security had to assist staff with applying restraints to a patient for medical reasons, such as a fall risk, pulling out tubes, or scratching wounds.
Assaultive	140 (1.5)	The patient was punching kicking, biting, spitting, or related. This would include incidents where officers or staff were injured by a patient. If the patient was restrained afterward, this code would not be used given that the incident would be documented as a patient restraint-aggressive.
Restraint-aggressive	430 (4.6)	Security had to assist staff with applying restraints to a patient due to the patient behaving in an aggressive manner.

### Data Analysis

The quantitative security log data and qualitative evaluation of clinical notes were initially analyzed separately, with thematic coders (L.T.S., A.S.B., E.K.G., and C.L.P.) blinded to the security log data and then compared and considered together for final conclusions. Hospital security log data are reported as numbers of encounters and proportions in each category from January 1, 2020, to December 31, 2021. Coding and analysis were done using Excel software Office 365 version (Microsoft Corp) on the subset of patient encounters that met inclusion criteria. Coders were blinded to the original study data, such as patient age, gender, and reason for mental health evaluation. Free-text snippets were deidentified prior to analysis, removing names of health care staff and any patient identifiers. A deductive analysis was performed to facilitate parsimony of coding and allow for expansion of codes, ensuring that all experiences were captured. An initial list of potential reasons for security involvement was generated based on clinical experiences of team members (L.T.S., A.S.B., E.K.G., and H.W.Y.) and relevant constructs from the literature (Table 2).^12,15,16,17^ Codes were nonexclusive. An *other* category was included to report additional actions or events. Each coder was trained on a dataset of 10 initial EHRs and then coded an additional 10 records under supervision (L.T.S.). The 2 original independent coders (A.S.B. and E.K.G.) are emergency medicine residents not involved in the parent study data abstraction. Coders with clinical experience were used given that this would allow them to better understand abbreviations and mannerisms in the clinical documentation. Remaining records were coded independently by 2 or more reviewers (L.T.S., A.S.B., and E.K.G.). Any EHRs without complete agreement were reviewed by a third, independent coder (C.L.P. or H.W.Y.). If the third coder agreed with a prior coder, that interpretation was used. If the third coder differed, the encounter went to the 5-coder committee and was discussed until consensus was reached.

**Table 2.** Initial Deductive Coding List and Definitions for Study of Documentation

Category code	Patients, No. (%) (N = 244)	Definition
Verbal threat to staff	72 (29.5)	Phrases referring to verbal threats to harm staff or others in the unit.
Physical aggression	82 (33.6)	Phrases that specify a kind of physical action (eg, fighting or kicking) that could hurt staff or other patients. Throwing items is included. Sexually aggressive behavior is also classified as a type of physical aggression. A clear event needs to be listed; the adjective “aggressive” is not enough.
Disagreement with care plan	41 (16.8)	Phrases referring to a competent patient refusing to adhere to a care plan or necessary care activities (eg, changing, taking medications, or discharge).
Treatment due to mental illness	27 (11.1)	Phrases referring to security assisting with immobilization for treatment or medication administration when the patient is unable to adhere due to mental illness.
Destruction of property	37 (15.2)	Includes walls, doors, computer equipment, clothing, or any other items. Throwing items or punching walls both qualify.
Verbal de-escalation by security	17 (7.0)	Phrases referring to de-escalation, calming down an agitated patient, or convincing a patient to adhere to treatment, done by security persons.
Verbal de-escalation by ED staff	29 (11.9)	Phrases referring to de-escalation, calming down an agitated patient, or convincing a patient to adhere to treatment, done by physicians, nurses, APPs, or other ED health care staff.
Transportation	81 (33.2)	Referring to transporting patients to or from any setting or place in the ED.
Patient retrieval or elopement	24 (9.8)	Phrases referring to searching for, chasing, or retrieving a patient who is trying to leave without permission.
Other	17 (7.0)	Additional events or actions that do not fit in the previously listed definitions.

Abbreviations: APP, advanced practice provider; ED, emergency department.

While there are many theories and frameworks evaluating the causes of violence in health care settings, this study was designed to delve into the third domain (organization) of a theoretical model of workplace violence, the Global Approach to Violence Toward Emergency Nurses.^18,19,20,21^ Most theories focus on patient, clinician, and environmental stressors, and only a few specifically address hospital security initiatives. This domain includes organizational approaches, including security protocols and teams, to limiting workplace violence. The predetermined coding strategy therefore began with coding the types of incidents when security was called to assist. This allowed us to somewhat match the way data are coded in hospital security logs and allowed for a content analysis approach. Once coded, data were sorted starting with condensed meaning units identified per code and then developing broader categories and, finally, themes. Themes and subthemes were discussed and agreed upon by the entire team during a group meeting. The study team included the head of hospital security (S.W.), an emergency physician and clinical researcher with experience in qualitative studies (L.T.S.), emergency medicine residents (A.S.B. and E.K.G.), a medical student (C.L.P.), and an emergency physician with a focus on diversity, equity, and inclusion (H.W.Y.). Data were analyzed from February to November 2024.

## Results

### Hospital Security Data

The hospital security team responded to 9288 clinical support calls for the ED over 2 years (mean, 12.7 encounters per day) (Table 1) among 139 872 university ED visits (6.6%). Demographics were not available given that these logs are collected in a different system than EHRs. Most support calls resulted in standby status, where no physical action was taken. Combining categories of verbally aggressive, threats, assaultive, and aggression requiring restraints, 737 hospital security encounters (7.9%) were for aggression.

The way security logs were coded meant that some security actions were not included in the security database as “clinical encounters.” Therefore the database we used did not include nonclinical encounters, such as “drug offense,” “no release of information” (NRI), “valuables pickup,” and “transportation escort.” This was discovered after comparing security log data with the qualitative analysis.

### Qualitative Analysis

Of 225 574 ED encounters in the 2-year time frame, 8512 encounters were for mental health needs and 334 patients were included in the study. Of those 344 patients, 244 patients (73.1%) had hospital security involved in their care and constituted the dataset for this qualitative analysis (146 men [59.8%]; mean [SD] age, 34.5 [13.6] years; 3 Asian American [1.2%], 116 Black [47.5%], 2 American Indian [0.8%], 95 White [38.9%], and 23 other race or multiracial [9.4%]; 4 Hispanic [1.6%]). The most frequent reason for calling security was physical violence (82 patients [33.6%]). There were 135 patients (55.3%) brought to the ED by police (Table 3). Most patients had prior mental health diagnoses (219 patients [89.8%]). The ED documentation ranged from very brief descriptions (“Pt [patient]punched nurse,” “security called,” “pt [patient] medicated”) to patient quotations and paragraph-long descriptions of incidents. “Pt” was frequently used as an abbreviation for patient in the documentation.

**Table 3.** Demographics of Patients in Emergency Department With Mental Health Concerns and Security Involvement

Characteristic	Patients, No. (%) (N = 244)
Age, mean (SD) [range], y	34.5 (13.6) [15-85]
Gender	
Women	95 (38.90)
Men	146 (59.84)
Transgender	2 (0.8)
Ethnicity	
Hispanic or Latino	4 (1.6)
Not Hispanic or Latino	240 (98.4)
Race	
Asian American	3 (1.2)
Black or African American	116 (47.5)
American Indian	2 (0.8)
White	95 (38.9)
Other or >1 race^a^	23 (9.4)
Missing	5 (2.0)
Brought in by police	135 (55.3)
Prior mental health diagnosis	219 (89.8)

^a^ Patients self-select the term multiracial at registration. Other race included Middle Eastern, Nepali, Somali, and unknown.

Agreement between initial coders ranged from 200 patients (81.9%) to 233 patients (95.6%) based on the code category. Interrater reliability was assessed using Cohen κ and was 0.76 for verbal threat, 0.65 for physical aggression, 0.40 for disagreement with care plan, 0.07 for unable to adhere due to mental health, 0.66 for destruction of property, 0.39 for verbal escalation by security, 0.37 for verbal escalation by staff, and 0.71 for transportation and retrieval or elopement. The assessment of whether a patient’s actions were due to disagreement with the care plan vs inability to adhere due to mental health reasons was the most common area of disagreement, with low κ values typically arising because 1 reviewer chose 1 category and the second reviewer chose the other category. Initial coders agreed completely for 97 patients (39.8%), while assessments for 121 patients (49.6%) were resolved by the third reviewer and assessments for 26 patients (10.7%) required committee discussion.

Most events were categorized into at least 1 initial code (212 patients [86.8%]) (Table 2). Additional reasons for the use of security personnel included patients being disruptive to others on the unit, security called to stop self-harming behaviors, and legal issues, such as disposal of illegal substances found in the person’s belongings and explaining NRI status to patients. A total of 15 patients (6.1%) had no clear reason in the text snippets for why security was involved in care.

### Coding of Themes

Subthemes are not the original codes. Subthemes were categories that arose from analyzing content units within original codes. For example, verbal threat to staff was an original code that fell under theme 1 of safety, but 2 subthemes of safety included patient-on-patient violence (a new determination for the ED) and timing of safety events (could occur at any point in the ED encounter). Neither of these were original codes but came from analyzing the content within codes.

#### Theme 1: Safety

Physical aggression occurred in one-third of patients receiving mental health care treatment (82 patients [33.6%]) where security was called to assist. This aggression included patient-on-staff violence, patient-on-patient violence, destruction of hospital and patient property, and sexually aggressive behaviors. Preventing self-harm was rarely seen.

##### Subtheme A: Types of Violence

Patient-on-patient violence and interruption of care for other patients was a novel finding, as was sexual violence and aggression.

Around this time this patient stood up out of her chair ran over to another patient and started punching another patient. (study identification [ID], 2135)

Pt screaming at staff, attempting to walk into other pt’s rooms. Pt interrupting other psych consults, difficult to redirect. Pt threatening this PCT [patient care technician], “I’ll f--- you up.” Pt raising fists. Security able to redirect pt to room but pt will come out minutes later yelling at staff again. (study ID, 1449)

We categorized sexual aggression as a form of physical violence. This included making threats of sexual violence against others or engaging in sexually explicit behaviors, such as refusing to stop masturbating or deliberately exposing genitalia.

Patient is now threatening to kill people, impregnating women. Having altercations with security officers. (study ID, 2298)

##### Subtheme B: Timing

Potentially unsafe events could occur at any time during the ED visit, from the initial evaluation to during routine medical care or during transport.

Patient brought to [ED] by ems [emergency medical services] screaming and thrashing on ems cot … Pt stating, “The next person to touch me I am going to punch in the face. I do not care what happens I will fight all of you at once. I do not care if I am restrained. I am ready to fight. I am ready to beat someone’s a--” Security present at bedside. Patient placed in 4 × restraints. Patient extremely violent, biting security, kicking, hitting and spitting at staff. (study ID, 407)

Security called to help obtain Covid swab. Pt bit RN [registered nurse] trying to obtain swab. Hallucinating. (study ID, 1792)

During patient change out from clothes to gown, patient is combative with security staff and bites security guard on the leg. (study ID, 268)

Pt calm in seclusion and taken to xray with security assistance. In xray pt became aggressive and security had to assist pt to remain free from harming staff. (study ID, 1583)

Safety also included being nearby if a need arose or in standby status. This was reflected in 5494 security calls (59.2%) and in the ED documentation.

Security called for standby as patient refusing to speak with staff but cooperative with IM [intramuscular] meds [medications]. Security on standby for update on plan for admission. Pt took information well and security intervention not required. (study ID, 1567)

#### Theme 2: Unable or Unwilling to Adhere to Care Plan

More than one-fourth of instances (68 patients [27.9%]) involved a patient who was unwilling or unable to adhere to the care plan. Sometimes patients refused medical care that was necessary, such as checking of vital signs or administering a COVID-19 swab, as in 1 quote previously stated. Similar to many other EDs, our ED places patients with suicidal or homicidal ideation in easily identifiable purple hospital gowns, and any items that could be used to harm the patients themselves or others are recorded and placed in secured lockers. Security teams assisted with these initial safety precautions for patients. Disposition from the ED was another frequent area of care plan disagreement. Patients who are a threat to themselves or others are sometimes involuntarily admitted to mental health facilities, and when informed of this, they may become upset, agitated, or aggressive.

Pt refusing to change into gown, yelling at staff and security. Pt eventually cooperative with changing into gown but remains irritable. Patient was combative with staff stating “I hope you[r] wife dies in a horrible way”. Pt is threatening to leave. Throwing chairs and ripped her IV out. Security assist RN with medication of patient. (study ID, 2382)

Pt continues to scream asking where her clothes are stating shes going to “AWOL this b----” pt yelling “I told that b---- earlier I’m not going to no psych hospital.” … Pt argumentative continues to states she will not go to [specific mental health facility] again informed pt that she cannot leave and the plan is to be admitted to [specific mental health facility]. (study ID, 121)

#### Theme 3: De-Escalation and Documentation of De-Escalation Attempts

Health care staff and security personnel are trained in verbal de-escalation of patients during mental health crises. Often, documentation did not fully discuss what types of de-escalation would be used or who would be performing these techniques. A total of 17 patients (7.0%) had encounters with documented de-escalation attempts from security, and 29 patients (11.9%) had encounters with documented de-escalation attempts from ED staff. Health care personnel often attempted de-escalation strategies prior to engaging hospital security, which may explain why the proportion of encounters with de-escalation by security was lower. Forms of de-escalation that were documented included redirecting the patient physically, redirecting the conversation, explaining the care plan, or reassuring patients of their safety.

Patient uncooperative with any direction to support/calm. Security at bedside. Pt refuses to sit down and becomes highly agitated/uncooperative with any attempt. Restrained with security and medicated. Patient escalating. (study ID, 76)

Dr. [name] at bedside to talk with pt about staying calm and she tried to punch her. Dr ordering meds. Security called and is present at bedside. They will stay for the administering of medication. Will continue to monitor. (study ID, 91)

Pt picked up stool in internal waiting room and threw it at consult room door. Security called to bedside, TW [this worker] and RN able to talk pt down at this time. Pt apologetic of his actions and pt educated about inappropriate behaviors previously displayed on unit. (study ID, 161)

#### Theme 4: Legal Concerns

Situations regarding legal concerns not only put the staff at risk but also create a greater liability for the hospital. Examples include illicit drug disposal, documentation of property, NRI status, and safe transportation. Safe transportation was included in the category of legal concerns because of the legal liability and concern for elopement during transportation of a patient under medical hold orders. The use of a security officer’s body camera to record the documentation of property was one novel use of hospital security.

Pt arrived to unit agitated and refusing to change security present at this time. Staff able to convince pt to change….While pt is changing TW sees pt put a medicine bottle into her underwear. TW notified security and after pt is finished changing TW goes into bathroom with pt and asks pt to remove medicine bottle from her underwear. Pt removes medicine bottle from underwear and medicine bottle is methadone. (study ID, 121)

[County police] already had belongings in bag with wallet, keys, cell phone, lighters. I had [hospital] security with body cam [camera] on while I counted his money. He had $15.00 cash and credit cards. I had [hospital] security stand by while I bagged and sealed the bag while the body cam was on. (study ID, 579)

Security at bedside to discuss NRI status. (study ID, 2244)

### Correlation of Qualitative and Quantitative Data

At a large-volume university hospital, hospital security was needed frequently, with a mean of 12.7 encounters per day but only 7.9% were for situations with aggression. This corresponds with our themes; while aggression was the most common concern in this patient subgroup that is at high risk, there were a variety of other reasons why security assistance was needed, including legal issues or being available on standby in case de-escalation measures were not successful. Situations of physical aggression requiring security assistance can happen anywhere in the ED and at any time in the patient visit. Our data suggest that these situations happened approximately daily in this large university hospital ED in 2020 to 2021. Documentation of these events is nonstandardized, and while security logs suggested that most events with patient aggression were de-escalated or resolved without force, the documentation in EHR notes did not often include de-escalation techniques used.

## Discussion

In this qualitative study with mixed methods analysis of the use of hospital security teams for patients receiving mental health care in the ED, security teams were integral to the safety of staff and patients. Security worked alongside the ED team in many different capacities, including safety, transportation, and legal documentation. In our subset of patients receiving mental health care in the ED, the most common reason for calling security was for physical aggression toward staff or other patients. While most clinical encounters in the ED overall were for standby assistance and did not result in physical force or restraints, most security encounters for patients receiving mental health care who required involuntary sedation involved aggressive or dangerous situations.

To our knowledge, this is the first study to compare health care team documentation and hospital security logs. While physical aggression was common in the subset of patients receiving mental health care, most security situations overall were resolved without the use of restraints. Restraints were used in 430 security encounters (4.3%). This is lower than what has previously been reported for use of restraints across all hospitalized patients (8.7%-11.8%), but these data include only restraint use when security was called.^22,23,24^ An analysis of the National Inpatient Sample database found that physical restraints were used in 0.63% of hospitalizations in the US, but this is likely an underestimate given that restraint use was categorized by payer codes instead of hospital documentation.^25^ Therefore, we suspect that our current use of hospital security and physical restraints in the ED is likely similar to those of other health systems. We encourage transparency from more hospitals in how they care for these patients at increased risk, which could help set standards and encourage improvement.

This qualitative analysis also identified 2 areas for improvement: reducing heterogeneity in clinical documentation and improving documentation of de-escalation. The security team has standardized documentation of each encounter, which is both written and visual (body cameras). However, our clinical teams currently do not have documentation standards. We noted different levels of detail and varying degrees of precision in clinical accounts. For example, some staff quoted patients word for word, while others used general statements, such as “making verbal threats.” Many events that require security assistance can be traumatizing or physically dangerous to patients. In certain instances, documentation of exact verbal threats may be necessary to explain why someone is being involuntarily held or medicated. However, overdocumentation of word-for-word quotations in patient care notes could be stigmatizing and bias clinicians or care. Patients may develop distrust of the medical community if they feel they are being ridiculed or singled out. EDs should consider developing an EHR template for security encounters or discussing expectations of what should be documented with staff.

Our next step is to assemble a team of ED staff, legal experts, security experts, and patient advocates to conduct thoughtful consideration of our documentation standards and goals. Our Workplace Safety Committee is also implementing standardized agitation documentation with an agitation scale. Agitation scales, while still prone to inaccuracy, help staff document behaviors and decide when to use different de-escalation techniques.^26,27^ Common agitation scales used in hospitals include the Richmond Agitation Sedation Scale, Behavioral Activity Rating Scale, and Agitated Behavior Scale.^28,29,30^

Our second focus is on improving de-escalation. Methods of de-escalation include providing a safe place, trying not to provoke, laying out clear expectations, listening to what the patient is saying, and offering choices.^31^ General phrases, such as “unable to be redirected” do not explain what was offered, how the patient perspective was included, or whether pain, anxiety, or fear were addressed.

### Limitations

Limitations to this study include that this was a secondary analysis of text snippets not abstracted for this purpose, which biases toward missing information. It is likely that the lower rates of documented de-escalation in this study were the result of how data were obtained, as snippets. Regardless, documentation templates or order sets could be used to help improve care and use of de-escalation techniques. Additionally, this is a single health system, and so our documentation and findings may not be generalizable.

## Conclusions

In this qualitative study that involved a mixed methods investigation of the use of hospital security during emergency mental health care, we found that hospital security teams frequently assisted in the ED and addressed violence and agitation but most situations were resolved without the use of physical restraints. Our study identified areas of opportunity in the ED. Given that safety threats are universally experienced in EDs, we encourage further research into ways to improve patient and staff safety and increased transparency in the use of security staff, restraints, and involuntary sedation in hospitals.

## Notes

Supplement.
Data Sharing Statement
