# Supplementary material for: Hospital Security Team Involvement in Emergency Mental Health Care of Patients
Source: JAMA Netw Open. 2025 Aug 29;8(8):e2530439. doi: 10.1001/jamanetworkopen.2025.30439 (PMC12397881; doi:10.1001/jamanetworkopen.2025.30439)
Supplement: Supplement. — Data Sharing Statement [file jamanetwopen-e2530439-s001.pdf]

Data Sharing Statement

Southerland. Hospital Security Team Involvement in Emergency Mental Health Care of Patients. *JAMA Netw Open*. Published online August 29, 2025. doi:10.1001/
jamanetworkopen.2025.30439
2025.27585

Data
Data available: No

Additional Information
Explanation for why data not available: The dataset contains sensitive information including quotes from patient charts and so is not approved by our hospital for sharing externally.
